# *Women’s Health Nursing*’s 30th anniversary: looking to the future

**DOI:** 10.4069/whn.2025.03.05.1

**Published:** 2025-03-28

**Authors:** Sukhee Ahn

**Affiliations:** College of Nursing, Chungnam National University, Daejeon, Korea

## Tracing the footsteps of *Women’s Health Nursing*

The Korean Society of Women Health Nursing was established independently in 1994 by the Korean Maternal and Child Nursing Association, which was established in 1970, and has made significant contributions to the development of education, research, and practice in women’s health nursing in Korea. With the establishment of our Society, the scope of women’s health nursing expanded beyond the scope of motherhood (pregnancy, childbirth, and postpartum) to include health care for women throughout their life span. As the Korean Society of Women Health Nursing celebrated its 30th anniversary in 2024, the society will continue to play a key role in advancing education, research, and practice in women’s health nursing in Korea.

*Women’s Health Nursing* (WHN), titled as *Korean Journal of Women Health Nursing* until 2023, is the official journal of the society. The journal published its first article in 1995, and we have now reached our 30-year milestone [1]. As former editor-in-chief, I have witnessed the journal’s pioneer role in scholarly publication among nursing journals, e.g., listed in the Korea Citation Index since 2005, Directory of Open Access Journals since 2013, CINAHL Plus since 2018, Scopus coverage since 2018, PubMed Central (PMC) and Emerging Sources Citation Index since 2020, and MEDLINE since 2023 [2]. The year 2025 marks the 30th anniversary of WHN, and we are preparing to publish our 31st volume. WHN will reflect our commitment to reaching a global audience and extending the journal’s scholarly contributions beyond Korea and Asia to authors worldwide. WHN is more committed than ever to disseminating women’s health research globally for nurses in academia and practice.

## Contemporary research topics

### Maternity and reproductive health

The most important research topics in women’s health nursing are issues related to the structure and function of the female reproductive system, including pregnancy, childbirth and birth, and reproductive health from menstruation to menopause. There is a wealth of descriptive and exploratory research in the field of reproductive health, with a focus on the structure and function of the female reproductive system, maternal and perinatal health care, and the exploration of disease adaptation and recovery in women and families experiencing reproductive health problems, e.g., cancer. What is needed now is more intervention research that allows accumulated women’s health nursing knowledge to move beyond describing, explaining, and predicting phenomena to prescribing interventions. To scientifically strengthen research, there is a need for more theoretically grounded research, practice-oriented research that can directly contribute to women’s health promotion, and evidence-based research that promotes evidence-based practice.

Consider the research needs in the field of women’s health nursing, especially in the context of ultra-low birth rates in Korea. In terms of women’s life cycle and reproductive health, motherhood is a normal life transition for women and families. We need to continue nursing research on women’s and families’ preparation for pregnancy and parenthood, healthy pregnancy, childbirth and parenting, and postpartum adjustment to parenthood as health care and demographic changes occur.

To ensure a global nursing perspective, there is also a need for contextual (e.g., country-based studies that include and illuminate sociocultural contexts) women’s health nursing research to inform evidence-based practice. Multinational collaborative research would also serve to improve the quality of maternal and women’s health care in line with global health standards.

The recent decline in birthrates in South Korea and Asia, as well as other countries around the globe, is not just a problem for women per se. Therefore, more research is needed to understand the declining fertility rate from a global perspective, including the promotion of women’s human rights and the autonomy to protect their sexual and reproductive rights, as well as the backlash against patriarchy and workplace culture and to develop legal and institutional improvements that enable work-life balance. Research into the risks of harm to women’s bodies and the instrumentalization of women’s bodies, particularly in relation to the increase in fertility treatments to increase reproductive opportunities due to low birth rates and the legal and ethical issues surrounding the promotion of human embryonic stem cell research should also be explored. There is also a continued need for research on sexually transmitted infections, miscarriages, and contraception, which have a long-term impact on women’s sexual and reproductive health.

### Gender-based women’s health issue

Caring for clients with health problems that are common to both sexes or with conditions that have gender differences in disease pathogenesis or prognosis is also essential for women’s health nursing research. This means more research is needed on the prevention and healthcare of gender-based physical, mental, and social health problems [3,4]. This is because nursing practice requires the application of gender-based health care strategies that take into account gender differences and the impact of gender differences on health problems that affect both men and women, such as depression, smoking, substance abuse, osteoarthritis, pain, coronary artery disease, osteoporosis, etc.

### Women’s rights and justice for reproductive health

Research is also needed on topics that can be addressed in the context of women’s human rights and reproductive health rights. These topics include menstruation, violence against women, the choice to stop menstruation, the right to terminate pregnancy, human trafficking, and inequalities in access to health care. Menstruation is linked to human dignity [5]. Without access to safe sanitation facilities and safe and effective menstrual products, menstruation cannot be managed with dignity. Menstrual exclusion, disregard, and discrimination also undermine the principle of human rights. As there are different sociocultural perspectives on menstruation around the world, it is necessary to look at issues related to women’s rights in relation to menstruation. Women’s health nursing researchers need to understand women’s bodies and health rights from a feminist perspective and work on issues to achieve justice in reproductive health.

### Health determinants affecting women’s health

Given the multiple factors influencing health status, more research is needed on the socioeconomic, environmental, social, and global factors determining women’s health beyond disease. This should include gender-specific women’s health research that considers perceptions of appearance, women’s forms of work, and care work in the home to improve women’s health. Understanding the unmet needs of special groups (e.g., teenage single mothers, older women, gender-expanding people) and the social and cultural contexts surrounding families is also necessary to intervene on their behalf.

## Future directions

WHN has proposed future initiatives to enhance the globalization of the journal [6]. Marking the 30th anniversary, I would like to suggest the following to further enrich future developments: broadening the purpose and scope of the journal, sharing more high-quality scientific and ethical papers that comply with international standard reporting guidelines, and increasing data sharing.

Most international journals covering women’s health focus on research across the life span of women, not just in a specific discipline but also through a multidisciplinary approach. Therefore, we need to extend a broader scope of research covering the entire lifespan of women, from puberty to older ages, and not just nursing but from all disciplines related to women’s physical, psychological, social, and cultural health issues globally that can appeal to an international audience. WHN must also publish high-quality content that applies to areas of education, research, and practice and disseminate it widely to enable evidence-based practice.

March 8 is International Women’s Day, a global day celebrating the social, economic, cultural, and political achievements of women. The day also marks a call to action for accelerating women’s equality with the “Accelerate Action” campaign theme in 2025 [7]. In light of this worldwide call to acknowledge strategies, resources, and activities that positively impact women’s advancement and to support and elevate their implementation [7], researchers need to do more ‘action’ research to ensure gender equality rights while exploring women’s bodies and health-related rights.

Our journal must make efforts to maintain an international level of academic excellence that secures ethical standards and scientific validity. For the scientific rigor of research, systematic research methods, logic, and consistency of statements are very important. To be scientifically valid, authors need to conduct theory-driven research, including a theoretical framework, and describe the theoretical framework in the manuscript. Research methods that are appropriate to the purpose and design of the study, a sample size that allows for adequate power, findings that are consistent with the purpose of the study, and a logical and valid discussion of the findings are essential. As thorough descriptions of such details are essential for readers to determine the scientific integrity of the study, authors are encouraged to be mindful of adhering to reporting guidelines in writing the manuscript. The journal has ascribed to internationally accepted reporting guidelines, e.g., the EQUATOR Network (https://www.equator-network.org/) since 2021. Specifying how research results can be applied to education, research, and practice in the region and globally to increase opportunities for dissemination of the body of knowledge will also be invaluable. We welcome submissions from international authors, especially studies reporting women’s health issues in Asia, as well as studies employing sex- and gender-based analysis.

Data sharing is strongly recommended to ensure transparency and replicability [6], and WHN supports this international standard, e.g., by utilizing the data repository, Harvard Dataverse (https://dataverse.harvard.edu), for authors who provide anonymized data.

In conclusion, WHN aims to be “a key resource for cutting-edge advancements and clinical applications of new nursing practice and innovative research on women’s health and nursing care.” By being indexed in PMC and MEDLINE, the WHN already reaches scholars and nurses around the world, providing valuable nursing knowledge and evidence for nursing practice and research. We seek continuous contributions from domestic and international researchers who submit, publish, and disseminate high-quality research related to women’s health nursing, which will enable the journal to continue its mission and aims and serve our readers-at-large.

## References

[1] Huh S (2023). Why do editors of local nursing society journals strive to have their journals included in MEDLINE?: a case study of the Korean Journal of Women Health Nursing. Korean J Women Health Nurs.

[2] Kim S (2024). Contributing to women's health nursing by disseminating research. Womens Health Nurs.

[3] Lee H (2022). Directions for sex and gender-based health research in Korea: implications of the Amendments of the Framework Act on Science and Technology. Korean J Women Health Nurs.

[4] Park H (2023). Women’s health care needs and what to consider. Health Welf Policy Forum.

[5] United Nations Population Fund (2022 May). Menstruation and human rights–frequently asked questions [Internet]. https://www.unfpa.org/menstruationfaq.

[6] Kim S (2022). Stepping stones for the future-2022 major developments, journal metrics, and appreciation for reviewers. Korean J Women Health Nurs.

[7] International Women’s Day IWD 2025 campaign theme is ‘Accelerate Action’ [Internet]. https://www.internationalwomensday.com/Theme.

